# Development of 65 Novel Polymorphic cDNA-SSR Markers in Common Vetch (*Vicia* *sativa* subsp. *sativa*) Using Next Generation Sequencing

**DOI:** 10.3390/molecules18078376

**Published:** 2013-07-16

**Authors:** Jong-Wook Chung, Tae-Sung Kim, Sundan Suresh, Sok-Young Lee, Gyu-Taek Cho

**Affiliations:** 1 National Agrobiodiversity Center, RDA, Suwon 441-853, Korea; 2 Department of Molecular Genetics, Ohio State University, Columbus, OH 43210, USA

**Keywords:** cDNA-SSR, genetic diversity, 454 sequencing, *Viciastiva* subsp. *sativa*

## Abstract

Vetch (*Vicia sativa* L.) is one of the most important annual forage legumes in the World due to its multiple uses (*i.e.*, hay, grain, silage and green manure) and high nutritional value. However, detrimental cyanoalanine toxins in its plant parts including seeds and its vulnerability to hard winter conditions are currently reducing the agronomic values of vetch varieties. Moreover, the existence in the public domain of very few genomic resources, especially molecular markers, has further hampered breeding efforts. Polymorphic simple sequence repeat markers from transcript sequences (cDNA; simple sequence repeat [SSR]) were developed for *Vicia* *sativa* subsp. *sativa.* We found 3,811 SSR loci from 31,504 individual sequence reads, and 300 primer pairs were designed and synthesized. In total, 65 primer pairs were found to be consistently scorable when 32 accessions were tested. The numbers of alleles ranged from 2 to 19, frequency of major alleles per locus were 0.27–0.87, the genotype number was 2–19, the overall polymorphism information content (*PIC*) values were 0.20–0.86, and the observed and expected heterozygosity values were 0.00–0.41 and 0.264–0.852, respectively. These markers provide a useful tool for assessing genetic diversity, population structure, and positional cloning, facilitating vetch breeding programs.

## 1. Introduction

*Vicia sativa* subsp. *sativa*, known as the common vetch, is one of the most commonly grown winter cover crops, or green manure, and is also used as pasture, silage, and hay [1,2]. It is cultivated with mixtures of cereal grains, providing cool-weather weed suppression and preventing fall N scavenging. It has been successfully applied in vineyards and orchards [1,2]. Due to its economic and ecological advantages, common vetch is now widespread through many parts of World, including the Mediterranean basin, west and central Asia, China, eastern Asia, India, and the USA [1,2,3]. 

The common vetch produces seeds that are quite similar to those of lentils in physical appearance and are highly nutritious [3,4,5,6,7]. However, due to the presence of cyanoalanine toxin in the seeds, which is detrimental to mono-gastric animals, including humans, the common vetch is currently tightly restricted as a feed or food source [3,4,5,6]. Moreover, its vulnerability to severe winter conditions (<−10 °C) further reduces its true agricultural potential [8,9]. Thus, to address these drawbacks, it is imperative to genetically improve this legume species either through conventional breeding or biotechnology approaches. However, a severe lack of genomic resources in the public domain has hampered such efforts.

Next-generation transcriptome sequencing is an excellent solution for enriching relevant genomic resources for non-model crop species such as the common vetch, providing functional annotations as well as genetic marker information [10,11,12]. In particular, cDNA-SSR markers generated from this approach can facilitate marker-assisted selection for vetch improvement programs, because these may be associated with functionally annotated transcribed genes, are cost-effective, and are easily transferable to related species [10,11,12,13]. Recently, we sequenced transcriptomes of common vetch using 454 pyrosequencing technology, and found 3,811 SSR loci from 31,504 individuals. In the present study, we developed and characterized polymorphic cDNA-SSR markers based on these transcriptome sequences to further contribute to breeding and molecular genetic studies of this species.

## 2. Results and Discussion

*V*. *sativa* subsp. *sativa* transcriptome sequencing yielded about 28 Mb and GS De Novo yielded 86,532 raw sequencing reads, based on the GS-FLX sequencer. SSRs are one of the most popular marker systems, consisting of various numbers of tandem-repeat di-, tri-, or tetra-nucleotide DNA motifs [14]. 

To identify SSR markers, we used the ARGOS program with default settings for *V*. *sativa* subsp. *sativa* singleton collections. In total, 3,811 potential SSR motifs were identified, with the majority being trinucleotide (76.3%) and dinucleotide (14.6%) repeats. There was a low rate (9%) of all other types of SSRs (e.g., tetra-, penta-, and hexa-nucleotide motifs) and the majority of trinucleotide SSRs had the GGT/GTG//TGG motif, followed by those with the ACC/CCA/CAC motif. In addition, CT/TC, AT/TA, and GA/AG motifs were abundant among the dinucleotide cDNA-SSRs. The relative proportion of SSR motif types was comparable to that of other plant species [15,16,17,18,19,20]. Kaur *et al.* [18] reported in theory, the frequencies of di-, tri-, tetra-, penta-, and hexanucleotide repeats should progressively decrease, based on the relative probability of replication slippage events. However, trinucleotide repeat units were predominant, followed by tetra-, di-, hexa-, and pentanucleotide repeat units.

Among the identified SSR loci, we selected 100 primer pairs on the basis of same annealing temperature, only 65 primer pairs produced single dominant polymerase chain reaction (PCR) products that were scorable for 32 accessions (Table 1 and Figure S1), The selected 65 polymorphic primer pairs sequences that were deposited in GenBank to provide a foundation for community genomic resources for vetch breeding and biotechnology research. The number of alleles (*N_A_*) per locus varied widely among the markers (Table 2) and ranged from 2 to 19, with an average of 6.6 alleles. The frequency of major alleles (*M_AF_*) per locus was 0.27–0.87 with an average of 0.508. In addition, the *H_O_* values were 0.00–0.86 with an average of 0.106, and the *H_E_* values were 0.264–0.852 with an average of 0.670. Lastly, polymorphic index content (*PIC*) values were 0.20–0.91, with an average of 0.59 Table 2. Considering the relatively high polymorphism levels, the cDNA-SSR markers developed in the present study will be useful for marker-assisted selection and population genetic studies to improve vetch varieties.

**Figure 1:**
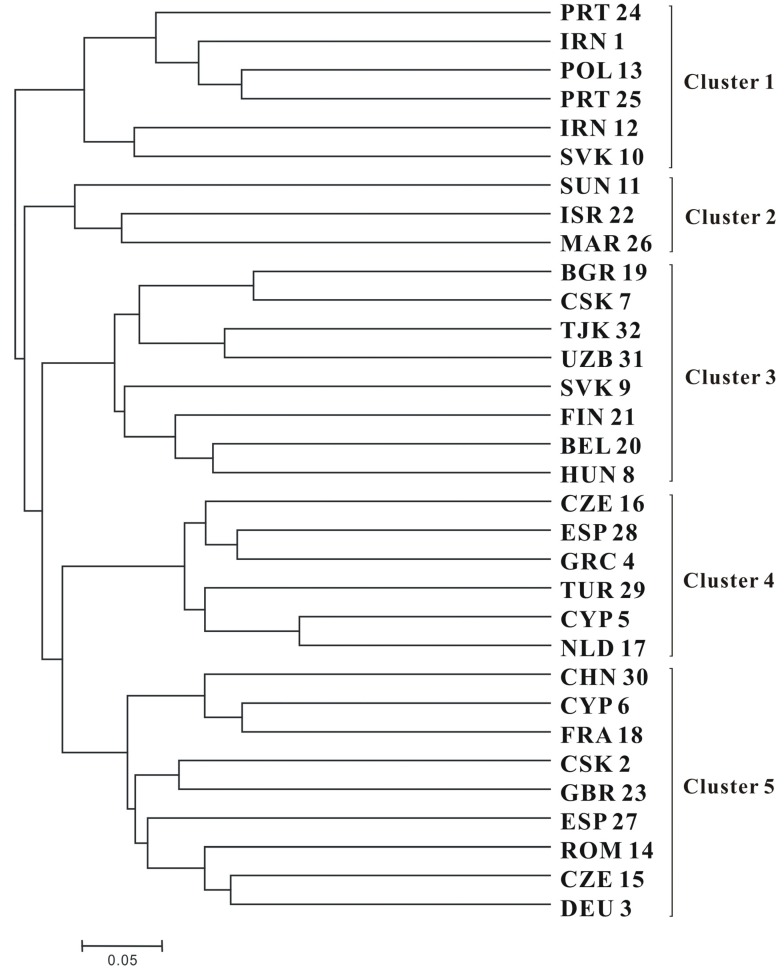
Dendrogram generated using UPGMA cluster analysis based on genetic diversity of 32 common vetch (*Vicia sativa* subsp. *sativa*) accessions.

The dendrogram showed that the 32 common vetch accessions fell into five distinct clusters (Figure 1). Cluster 1 comprised accessions from South west Europe, West Asia and Central Europe each regions had 2 accessions. Cluster 2contained 3 accessions one from Eurasian, one from middle East Asia and one from North Africa. Cluster 3 contained eight accessions, five accessions from Central Europe and remaining accessions from South East Europe, north Europe and West Europe each region had one accessions. Cluster 4 contained six accessions, two accessions from South East Europe and one accession from each geographical region (Central Europe, South West Europe, Europe and North West Europe). Cluster 5 included nine accessions, two accessions from central Europe and two accessions from Europe regions, with rest of the accessions from East Asia, West Europe, South West Europe, Central and South East Europe and West General Europe, North Europe and West Europe each having one accession.

Dongi *et al* [21] reported cluster analysis of *Trigonella foenumgraecum* there was no clear clustering pattern of geographically closer accessions indicating that the association between genetic similarity and geographical distance was less significant. However, it is necessary to use more number of accessions from each geographical location to confirm the available pattern.

**Table 1:** Chaμracteristics of the 65 cDNA-SSR markers for common vetch (*Vicia sativa* subsp. *sativa*).

Marker	Primer sequence (5'-3')	Motif	GenBank Acc. No.	Ta (°C)	BLAST top hit Acc. No.	Description	E-value
GBSSR-VSspS-020	F: CATTTGGCTGATCCTGTCA R: GGCTTCATCATGAGACAAGAA	(TAT)5	KF008486	55	L19651.1	*Pisum sativum* chloroplast photosystem I 24 kDa light harvesting protein (lhca3) mRNA, complete cds	7e-92
GBSSR-VSspS-023	F: CCTGCATTCACAACCATTT R: CGCCATCGATGTTTTGTT	(CAT)5	KF008487	55	None	None	None
GBSSR-VSspS-024	F: ACGGTGTTAACGGTCACG R: TCTCCAAACCGACACCAG	(AGA)5	KF008488	55	AF262939.1	*Pisum sativum* chloroplast protein import component Toc159mRNA. complete cds	1e-160
GBSSR-VSspS-028	F: TGAGCCGTTGACACAACA R: GGCGATCCTCCTACTTGAA	(TGC)5	KF008489	55	None	None	None
GBSSR-VSspS-037	F: GAAACAAGCTGAAGGCCC R: TCAGGAAATGACCAAACCA	(GAA)5	KF008490	55	X76774.1	*P.sativum* mRNA for HMG1 protein	2e-123
GBSSR-VSspS-038	F: CTCCCCAACTTGTTCCCT R: GGGAACTTGTCGATGTGG	(TTC)5	KF008491	55	BT143793	*Medicago truncatula* clone JCVI-FLMt-3P13 unknown mRNA	3e-41
GBSSR-VSspS-042	F: GGTTCGAGAGCTTTGCTG R: CTGTGCCACTTGACCTCC	(CGT)5	KF008492	55	JF965421.1	*Medicago sativa* ethylene response factor 11 (ERF11) mRNA, complete cds	2e-37
GBSSR-VSspS-057	F: GAGGTTTCCGGTGAGGAG R: GTTCCAGCAGGTGAAGCA	(GGT)7	KF008493	55	XM_003548374.1	PREDICTED: *Glycine max* DEAD-box ATP-dependent RNA helicase 31-like (LOC100799999), mRNA	7e-28
GBSSR-VSspS-066	F: AGGAGAGGCAAGGACCAG R: CACGGCTATTTTCTTCTTTTTC	(GA)10	KF008494	55	AB078603.1	*Pisum sativum* SN4TDR mRNA for 110kDa 4SNc-Tudor domin protein, complet cds	1e-93
GBSSR-VSspS-067	F: CAAACTTGTCACCACATATACAA R: GGTGGTCACTAGTGGAGGTG	(CCA)5	KF008495	55	KC218790.1	*Vicia faba* clone NAC-VF- 218 microsatellite sequence	3e-39
GBSSR-VSspS-071	F: GTATTCCTCTGGTGGTGGG R: CACCACCAAGACCTCCAA	(TGG)5	KF008496	55	XM 003617084.1	*Medicago truncatula* Heterogeneous nuclear ribonucleoprotein DO (MTR 5g088220) mRNA, complete cds	9e-108
GBSSR-VSspS-073	F: CCTCCCAATCCTCCATTC R: CCCTAGTCCTCCAATTTCG	(GTT)5	KF008497	55	XM_004506417.1	PREDICTED: *Cicer arietinum* scarecrow-like protein 6-like (LOC101497219), mRNA	2e-58
GBSSR-VSspS-075	F: TTCAGCAAGCCCATCATT R: CGTCCGTCCAATCAACAA	(TTA)6	KF008498	55	JF768700.1	*Lens culinaris* microsatellite LcSSR535 sequence	1e-71
GBSSR-VSspS-076	F: CCTGGTCCCAGAAATGGT R: AAGCCAGAGGGCATTGAT	(CTG)6	KF008499	55	XM_003602555.1	*Medicago truncatula* NAC domin protein (MTR_3g096140) mRNA complete cds	1e-136
GBSSR-VSspS-079	F: AAAGCAAATTGTTAAAGAAAGGG R: GAGGATGCTGCACATATGTAGTT	(AAT)5	KF008500	55	None	None	None
GBSSR-VSspS-080	F: AATGCATGGATCGAGGTG R: GAATCCATCGGCAACGTA	(TGG)5	KF008501	55	XM_004510745.1	PREDICTED: *Cicer arietinum* uncharacterized LOC 101512367 (LOC 101512367), transcript variant X3, mRNA	6e-110
GBSSR-VSspS-088	F: CGAAGAGGTAAATGACGCC R: AGTGACCTATATTTAGCATCGTT	(TGG)5	KF008502	55	None	None	None
GBSSR-VSspS-090	F: AGACGCACCACAACAGAAA R: GGGCTAGACATGGCACAA	(AGC)6	KF008503	55	BT149661.1	*Medicago truncatula* clone JCVI-FLMt-1507 unknown mRNA	7e-62
GBSSR-VSspS-091	F: CCAAACCAGCAAGAGCAG R: GAGCAGCGTTGTCTCGTC	(CTT)5	KF008504	55	XM_003594377.1	*Medicago trancatula* Endoglucanase (MTR_2g028480) mRNA complete cds	e-122
GBSSR-VSspS-099	F: ATCCATGCCTCTTTTGCC R: AGCCTCATTTCAGCAGCA	(TCT)5	KF008505	55	BT137674.1	*Medicago truncatula* clone JCVIMt-1708 unknown mRNA	3e-77
GBSSR-VSspS-102	F: TTCAACGGAGATGGATCG R: CGTCTTCTTTCAGAGGCG	(GTT)5	KF008506	55	X59773.1	*Pisum sativum* mRNA for P protein. a part of glycine cleavage complex	0.0
GBSSR-VSspS-107	F: TGGTTTCTTTCTAAAGGGGTG R: CGGCTCGATGGACAGTAG	(GTT)5	KF008507	55	BT146949.1	*Medicago truncatula* clone JCVI-FLMt-19I1 unknown	e-127
GBSSR-VSspS-115	F: CATAAACAAGGGCAAGAAAA R: GAGGAAAACATTGGTGGGA	(TGC)6	KF008508	55	XM_004501895.1	PREDICTED: *Cicer arietinum* nucleobase- ascorbate transporter 6-like (LOC101504609), transcript X2, mRNA	6e-59
GBSSR-VSspS-117	F: CGGTGCACTAAGTGGGAA R: TTAATGATGGTGGCGAGG	(AGG)5	KF008509	55	BT135350.1	*Medicago truncatula* clone JCVI-FLMt-9H4 unknown mRNA	2e-114
GBSSR-VSspS-118	F: GCATTTCCCTTGGTCTCC R: CAGAAAGAGCAACCGTGC	(TGG)5	KF008510	55	XM_004514512.1	PREDICTED: *Cicer arietinum* N-alpha-acetyltransferase 10-like (LOC101504041), mRNA	3e-68
GBSSR-VSspS-119	F: CACCACCAAGACCTCCAA R: CCATCATCATCACCAGCC	(ACC)5	KF008511	55	XM_003617084.1	*Medicago truncatula* Heterogeneous nuclear ribonucleoprotein D0 (MTR_5g088220), complete cds	9e-98
GBSSR-VSspS-125	F: GGCCGGTATTCGTCAACT R: CCCCGTATTTTCTCGGTC	(TGG)5	KF008512	55	XM_004510745.1	PREDICTED: *Cicer arietinum* uncharacterized LOC 101512367 (LOC101512367), transcript variant X3, mRNA	2e-134
GBSSR-VSspS-126	F: TGGCGCTTATCGCTATGT R: TCCACTCATTCCACTCGT	(TG)7	KF008513	55	XM_003623363.1	*Medicago truncatula* Patellin-6 (TR_7G070480) mRNA, complete cds	5e-81
GBSSR-VSspS-129	F: AGGAGAGGCAAGGACCAG R: CTTTTTCTCTAACTCATTCATGTC	(GA)10	KF008514	55	AB0786031	*Pisum sativum* SN4TDR mRNA for 110kDa 4SNc-Tudor domain protein, complete cds	8e-117
GBSSR-VSspS-135	F: TGGTGGAGATTTGTTGGG R: CTTCATCTTCCCACACCG	(TGG)5	KF008515	55	BT136030.1	*Medicago truncatula* clone JCVI-FLMt-16L14unknown mRNA	4e-40
GBSSR-VSspS-138	F: CGGAGTTCACATAAAACATACTAC R: TGGGAGTGTTGAGATGGG	(TTA)7	KF008516	55	AB176563.1	*Vicia faba* MET mRNA for type 2 metallothionein, complete cds	6e-98
GBSSR-VSspS-140	F: TTGCTTTGATGTTTGGAGC R: CCCTAAATTCCCAACCCA	(GGT)7	KF008517	55	XM_003613856.1	*Medicago truncatula* Cysteine-rich receptor-like protein kinase (MTR_5g042440) mRNA, complete cds	3e-86
GBSSR-VSspS-156	F: GGCCAATTTAGCGAGCTT R: CACTATCATCAACCTCTAACGGA	(GTG)5	KF008519	55	BT134176.1	*Medicago truncatula* clone JCVI-FLMt-15D24 unknown mRNA	2e-118
GBSSR-VSspS-158	F: TGAGCTTATTGCCAGTGGA R: CCATGTCATCATCGGATTC	(TGG)5	KF008520	55	KC218603.1	*Vicia faba* clone NAC-VF-31 microsatellite sequence	7e-124
GBSSR-VSspS-162	F: GAGACAGTGGAAGTATCGGC R: CACAGCAAATGCATCGGT	(AAG)6	KF008521	55	BT146412.1	*Medicago truncatula* clone JCVI-FLMt-21014 unknown mRNA	1e-75
GBSSR-VSspS-166	F: GTGGCCATGATCCATTTG R: TTCCTCGAGAGGGAAAGC	(TGG)5	KF008522	55	XM_003605932.1	*Medicago truncatula* DnaJ (MTR_4g50420) mRNA complete cds	0.0
GBSSR-VSspS-172	F: GCTTTGGAAGAGCCCAAT R: TCCAGGATTGTAACCCCC	(TGG)5	KF008523	55	XM_003617084.1	*Medicago truncatula* Heterogeneous nuclear ribonucleoprotein D0 (MTR_5g088220) mRNA, complete cds	5e-90
GBSSR-VSspS-173	F: GGGCACGGTGGTCACTA R: TGACTACCACCACCTCCG	(TGG)5	KF008524	55	AJ831469.1	*Pisum sativum* mRNA for putative glycine rich protein precursor (grp1 gene)	2e-13
GBSSR-VSspS-179	F: AGCTATGCGAGAGGCTCC R: CTGTGGGAAGGCACATCT	(TGA)6	KF008525	55	None	None	None
GBSSR-VSspS-181	F: CACTGTGACTCAGTTTCGTTG R: CGATTTTGAACCCTAACCG	(TTC)5	KF008526	55	None	None	None
GBSSR-VSspS-182	F: GCGTTGTGGCGTATTTCT R: TGGAGGAAAGGAAACTACTCA	(GCA)6	KF008527	55	AB676029.1	*Lathyrus japonicas* DNA, 61 locus, haplotype: D	4e-62
GBSSR-VSspS-185	F: CTCCTCAATTTTCCCCCA R: TTTGGTGCGATTGTTTCC	(CAT)5	KF008528	55	Ap009676.1	*Lotus japonicas* genomic DNA, clone: LjT30I08, TM0492	4e-26
GBSSR-VSspS-187	F: CCAGGTTGCTTTCCTTACTTT R: TTAGCCCTCAAAGCCTCC	(ATC)5	KF008529	55	X54359.1	*P.sativum* mRNA of cDNA clone 26g	2e-154
GBSSR-VSspS-192	F: AGGGTCTTCCTTCCCACA R: TATGGTGACACGTTCGCA	(ATC)5	KF008530	55	XM_004505980.1	PREDICTED: *Cicer arietinum* uncharacterized LOC101500025 (LOC101500025), transcript variant X2, mRNA	1e-100
GBSSR-VSspS-203	F: TCCATCTGGTTGGTGGTG R: GAAAGCCAATTTTTCAGCAA	(GTT)7	KF008531	55	BT147294.1	*Medicago truncatula* clone JCVI-FLMt-15A20 unknown mRNA	2e-50
GBSSR-VSspS-217	F: CCATCGCCACCACCA R: TCCCGGAACAAAAATCAA	(AAC)7	KF008532	55	EF447278.1	*Pisum sativum* cultivar Finale SYM8 (SYM8) gene, partial cds	5e-58
GBSSR-VSspS-245	F: CAATAGGGGGACCCTTCA R: GCTGCAAGCTGCTACCAT	(GGA)5	KF008533	55	HQ439603.1	*Phalaenopsis* hybrid cultivar candidate developmental transcription factor TCP1 mRNA, partial cds	5e-04
GBSSR-VSspS-247	F: GGTTCAATACGATCCATAGAATA R: TGATCGCCAATTCTGGAC	(CAC)6	KF008534	55	KC294548.1	*Aeschynomene ciliate* voucher IRRI 13078 cyclophilin 1 (CYP1) gene, complete cds	3e-46
GBSSR-VSspS-249	F: AAAACATGGTTGAGTGTTTTTG R: TAACCCTCTCGGTTTCGG	(ATA)5	KF008535	55	KC218749.1	*Vicia faba* clone NAC-VF-177 microsatellite sequence	2e-55
GBSSR-VSspS-251	F: TGGTGGACGTCACTATGGA R: CATGGTGCTTCCGACAAT	(TGG)5	KF008536 *	55	KC218790.1	*Vicia faba* clone NAC-VF-218 microsatellite sequence	5e-30
GBSSR-VSspS-252	F: CATGGTGCTTCCGACAAT R: TCGAAATCAGGACTTACCACA	(CCA)5	KF008536 *	55	KC218790.1	*Vicia faba* clone NAC-VF-218 microsatellite sequence	5e-30
GBSSR-VSspS-262	F: ATTGGGCCCTCTTTTTGA R: GGGGGTAGAAAAGTTGCG	(AT)7	KF008537	55	XM_004500781.1	PREDICTED: *Cicer arietinum* dolichyl- diphoshooligosaccharide-protein glycosyltransferase subunit 1A-like (LOC101502563), mRNA	1e-37
GBSSR-VSspS-268	F: AAATTTGTCTGACGAAAAACG R: TGCTTGAGAGTGCCATCA	(TAC)5	KF008538	55	BT144509.1	*Medicago truncatula* clone JCVI-FLMt-13M6 unknown mRNA	2e-47
GBSSR-VSspS-269	F: TTCCATTTATCCTCCTATCCTCT R: CTTGAATGCGAAACGAGG	(CGC)5	KF008539	55	XM_004507695.1	PREDICTED: *Cicer arietinum* eukaryotic translation initiation factorisoform 4G-1-like (LOC101492356), mRNA	1e-56
GBSSR-VSspS-284	F: TGGAAGGAAATGGCAGTG R: ATCCGTTTCGGATTGGTT	(GCA)5	KF008540	55	JX539287.1	*Vigna radiata* cultivar MCV-1 clone GGSSR_911 microsatellit sequence	2e-120
GBSSR-VSspS-291	F: CCCAACCGAACCACTTATT R: TAATAGCTCCGGCCCAGT	(CTA)5	KF008541	55	None	None	None
GBSSR-VSspS-301	F: AACCAAACAACAATGGGTT R: TCAACCGGTGAAAGATGG	(CAA)5	KF008542	55	JN849865.1	*Medicago falcata* voucher PI494662A caffeic acid-O- methyltransferase (COMT) gene.exon 1 and partial cds	2e-128
GBSSR-VSspS-304	F: CCGTTCTACGCAATTCTCC R: CGACCAAGAACACCAGGA	(TTC)5	KF008543	55	XM_003593951.1	*Medicago truncatula* hypothetical protein (MTR_2g020190) mRNA,complete cds	1e-55
GBSSR-VSspS-305	F: CATGAAAGAGTTTTGCACCTT R: CCGACGACGAGATTGAGA	(GCA)5	KF008544	55	None	None	None
GBSSR-VSspS-308	F: TGAGAGCATAGACAGCAAACA R: TGGATTTGGTCGCATAGC	(AAC)5	KF008545	55	None	None	None
GBSSR-VSspS-309	F: TCTTCAAAAGAGTACAAAAGGGA R: GAATTGGACACCTTGGCA	(AAT)5	KF008546	55	BT137399.1	*Medicago truncatula* clone JCVI-FlMt-19L22 unknown mRNA	5e-124
GBSSR-VSspS-310	F: GGGTGCCCTAGCATTTGT R: ATCTCCGGCGTCAGTTTC	(CTC)6	KF008547	55	M69105.1	*Pisum sativum* outer membrane protein (OM14) mRNA, complete cds	9e-96
GBSSR-VSspS-311	F: TTGAGGCGGTGTTGGTAG R: ATGTCATGGCCAACTGCT	(GGA)6	KF008548	55	None	None	None
GBSSR-VSspS-313	F: GAACAATGCAGCCTGGAA R: GCTGCAATCGCATTCTCT	(TTG)5	KF008549	55	XM_003613196.1	*Medicago truncatula* U-box domin containing protein (MTR_5g034440) mRNA, complete cds	2e-112

TA, annealing temperature. * same sequence two primers identified.

**Table 2:** Diversity statistics from initial primer screening in 32 accessions of common vetch (*Vicia sativa* subsp. *sativa*).

Marker	*N_A_*	*M_AF_*	*H_O_*	*H_E_*	*PIC*
GBSSR-VSspS-020	4	0.63	0.00	0.51	0.43
GBSSR-VSspS-023	7	0.34	0.00	0.75	0.71
GBSSR-VSspS-024	7	0.47	0.00	0.71	0.67
GBSSR-VSspS-028	4	0.38	0.00	0.73	0.68
GBSSR-VSspS-037	4	0.38	0.00	0.71	0.65
GBSSR-VSspS-038	7	0.28	0.00	0.79	0.76
GBSSR-VSspS-042	2	0.84	0.00	0.26	0.23
GBSSR-VSspS-057	8	0.36	0.03	0.77	0.74
GBSSR-VSspS-066	7	0.25	0.00	0.83	0.81
GBSSR-VSspS-067	6	0.50	0.00	0.67	0.62
GBSSR-VSspS-071	3	0.50	0.00	0.62	0.54
GBSSR-VSspS-073	8	0.31	0.00	0.81	0.79
GBSSR-VSspS-075	6	0.38	0.03	0.76	0.72
GBSSR-VSspS-076	3	0.56	0.00	0.57	0.50
GBSSR-VSspS-079	6	0.55	0.00	0.64	0.60
GBSSR-VSspS-080	3	0.50	0.00	0.59	0.51
GBSSR-VSspS-088	5	0.38	0.00	0.74	0.70
GBSSR-VSspS-090	7	0.41	0.00	0.75	0.71
GBSSR-VSspS-091	4	0.47	0.00	0.68	0.64
GBSSR-VSspS-099	4	0.56	0.00	0.60	0.54
GBSSR-VSspS-102	8	0.44	0.03	0.75	0.73
GBSSR-VSspS-107	3	0.66	0.00	0.51	0.45
GBSSR-VSspS-115	4	0.48	0.03	0.54	0.44
GBSSR-VSspS-117	6	0.41	0.00	0.71	0.67
GBSSR-VSspS-118	3	0.53	0.00	0.61	0.54
GBSSR-VSspS-119	2	0.65	0.00	0.46	0.35
GBSSR-VSspS-125	3	0.41	0.00	0.63	0.56
GBSSR-VSspS-126	7	0.25	0.00	0.82	0.79
GBSSR-VSspS-129	7	0.25	0.00	0.81	0.79
GBSSR-VSspS-135	5	0.50	0.00	0.62	0.55
GBSSR-VSspS-138	11	0.32	0.06	0.83	0.81
GBSSR-VSspS-140	8	0.22	0.03	0.81	0.79
GBSSR-VSspS-151	5	0.67	0.03	0.52	0.48
GBSSR-VSspS-156	5	0.66	0.00	0.53	0.50
GBSSR-VSspS-158	5	0.59	0.00	0.57	0.52
GBSSR-VSspS-162	6	0.53	0.00	0.66	0.63
GBSSR-VSspS-166	6	0.38	0.00	0.74	0.70
GBSSR-VSspS-172	3	0.41	0.00	0.65	0.57
GBSSR-VSspS-173	4	0.58	0.00	0.58	0.53
GBSSR-VSspS-179	8	0.56	0.03	0.64	0.61
GBSSR-VSspS-181	11	0.28	0.00	0.85	0.84
GBSSR-VSspS-182	7	0.38	0.00	0.76	0.73
GBSSR-VSspS-185	10	0.31	0.03	0.82	0.80
GBSSR-VSspS-187	5	0.44	0.00	0.69	0.64
GBSSR-VSspS-192	3	0.63	0.00	0.51	0.43
GBSSR-VSspS-203	6	0.34	0.00	0.76	0.72
GBSSR-VSspS-217	11	0.28	0.00	0.83	0.81
GBSSR-VSspS-245	7	0.28	0.00	0.81	0.78
GBSSR-VSspS-247	6	0.69	0.03	0.49	0.46
GBSSR-VSspS-249	6	0.56	0.00	0.64	0.61
GBSSR-VSspS-251	5	0.45	0.00	0.70	0.66
GBSSR-VSspS-252	3	0.50	0.00	0.61	0.53
GBSSR-VSspS-262	9	0.47	0.00	0.72	0.70
GBSSR-VSspS-268	3	0.47	0.00	0.64	0.57
GBSSR-VSspS-269	4	0.50	0.00	0.58	0.49
GBSSR-VSspS-284	6	0.44	0.00	0.66	0.60
GBSSR-VSspS-291	5	0.53	0.00	0.64	0.59
GBSSR-VSspS-301	3	0.53	0.00	0.58	0.50
GBSSR-VSspS-304	7	0.46	0.00	0.68	0.63
GBSSR-VSspS-305	3	0.69	0.00	0.48	0.43
GBSSR-VSspS-308	6	0.54	0.00	0.66	0.63
GBSSR-VSspS-309	8	0.34	0.00	0.78	0.75
GBSSR-VSspS-310	8	0.38	0.03	0.75	0.71
GBSSR-VSspS-311	7	0.44	0.00	0.73	0.70
GBSSR-VSspS-313	5	0.44	0.00	0.71	0.67
Mean	5.7	0.460	0.006	0.670	0.624

*NA*, number of alleles; *MAF*, major allele frequency; *HO*, observed heterozygosity; *HE*, expected heterozygosity; *PIC*, polymorphic information content.

## 3. Experimental

### 3.1. Plant Material

*Vicia sativa sativa* seeds were selected from the National Agrobiodiversity Center, Rural Development Administration, Suwon, Korea (Table 3). Seedlings were germinated and grown in a glasshouse. The leaves of young seedlings were used to extract the mRNA required to synthesize the cDNA library and for 454 sequencing.

**Table 3:** List of common vetch (*Vicia sativa* subsp. *sativa*) accessions.

No.	Temp. ID	USDA-ARS No.	Country of origin	Geographical region of origin
1	K193581	PI 226487	Iran	West Asia
2	K193582	PI 284058	Czech Republic	Central Europe
3	K193583	PI 284068	Germany	Western Central Europe
4	K193584	PI 284078	Greece	South East Europe
5	K193585	PI 284402	Cyprus	Europe
6	K193586	PI 284409	Cyprus	Europe
7	K193587	PI 284470	Czech Republic	Central Europe
8	K193588	PI 284471	Hungary	Central Europe
9	K193589	PI 308111	Slovakia	Central Europe
10	K193590	PI 308118	Slovakia	Central Europe
11	K193591	PI 325513	Soviet Union	Eurasian
12	K193592	PI 381065	Iran	West Asia
13	K193593	PI 393870	Poland	Central Europe
14	K193594	PI 393871	Romania	Central and South East Europe
15	K193595	PI 393872	Czech Republic	Central Europe
16	K193596	PI 393873	Czech Republic	Central Europe
17	K193597	PI 393877	Netherlands	North West Europe
18	K193598	PI 393878	France	West Europe
19	K193599	PI 393891	Bulgaria	South East Europe
20	K193600	PI 393904	Belgium	West Europe
21	K193601	PI 393907	Finland	North Europe
22	K193602	PI 393909	Israel	Middle East Asia
23	K193603	PI 393910	United Kingdom	Europe
24	K193604	PI 393912	Portugal	South West Europe
25	K193605	PI 493307	Portugal	South West Europe
26	K193606	PI 517191	Morocco	North Africa
27	K193607	PI 533741	Spain	South West Europe
28	K193608	PI 533743	Spain	South West Europe
29	K193609	PI 557498	Turkey	South East Europe
30	K193610	PI 577751	China	East Asia
31	K193611	PI 628288	Uzbekistan	Central Europe
32	K193612	PI 664293	Tajikistan	Central Europe

(Temp ID), Korean GeneBank ID; (ARS No.), USDA-ARS Number.

### 3.2. cDNA Preparation

Total RNA was extracted from *Vicia sativa* subsp. *sativa* leaves that were frozen in liquid nitrogen, ground into a powder, and then extracted using an RNeasy Plant Mini kit (Qiagen, Valencia, CA, USA) following the manufacturer’s instructions. The integrity of total RNA was determined using a BIOSPEC-NANO spectrophotometer (Shimadzu, Kyoto, Japan) and agarose gel electrophoresis. mRNA was purified using the PolyATract mRNA Isolation System IV (Promega, Madison, WI, USA), and the purified products were used to synthesize full-length cDNAs using a ZAP-cDNA Synthesis kit (Stratagene, Santa Clara, CA, USA). Finally, cDNA was fragmented by nebulization for library construction.

### 3.3. Library Preparation

Approximately 1 µg cDNA was used to generate a DNA library to use with the Genome Sequencer GS-FLX Titanium System (Roche, 454 Life Science, Branford, CT, USA). The cDNA fragment ends were polished (blunted), and two short adapters were ligated to both ends according to standard procedures described previously**.** The adapters, along with the sequencing key, a short sequence of four nucleotides used by the system’s software for base calling, provided priming of the sequences for both the amplification and sequencing of the sample library fragments. Following the repair of any nicks in the double-stranded library, the unbound strand of each fragment was released (with 5-Adaptor A). Finally, the quality of this single-stranded template DNA library was assessed using a 2100 BioAnalyzer (Agilent, Waldbronn, Germany). The library was quantified to determine the optimal amount needed as input for emulsion-based clonal amplification.

### 3.4. 454 Pyrosequencing

Single effective copies of template species from the DNA library to be sequenced were hybridized to DNA capture beads. Then the immobilized library was resuspended in an amplification solution, and the mixture was emulsified, followed by PCR amplification. The DNA-carrying beads were recovered from the emulsion and enriched after amplification. The second strands of the amplified products were melted, leaving the amplified single-stranded DNA library bound to the beads. Then the sequencing primer was annealed to the immobilized amplified DNA templates. After amplification, a single DNA-carrying bead was placed into each well of a PicoTiterPlate (PTP) device. Simultaneous sequencing with multiple samples on a single PTP (four-region gasket) was used. Then the PTP was inserted into the FLX Genome Titanium sequencer for pyrosequencing [22,23], and sequencing reagent was flowed sequentially over the plate. Information from the PTP wells was captured simultaneously by a camera, and the images were processed in real-time by an onboard computer. Multiplex identifiers were used to specifically tag unique samples in a GS FLX Titanium sequencing run, which were recognized by the GS data analysis software after the sequencing run and provided high confidence for assigning individual sequencing reads to the correct sample. Sequence assembly was performed after sequencing using GS De Novo Assembler software (Roche) to produce contigs and singletons. All sequence data were conformed to references using GS Reference Mapper software (Roche). 

### 3.5.Discovery of cDNA-SSR Markers

All contigs and singletons from both transcriptomes were used to mine SSR motifs, and SSR motifs were identified using the ARGOS pipeline program (version 1.46) at the default settings to survey the molecular markers present in the *V*. *sativa* subsp. *sativa* accessions**.** Parameters were designed for identifying perfect di-, tri-, tetra-, penta-, and hexa-nucleotide motifs with a minimum of six repeats. The primer design parameters were set as follows: length range, 18–23 nucleotides with 21 as optimum; PCR product size range, 100–400 bp; optimum annealing temperature, 55 °C; and GC content 40–60%, with 50% as optimum. *Vicia sativa* subsp. *sativa* genomic DNA was extracted from 32 diverse common vetch accessions for cDNA-SSR marker validation using a DNeasy® Plant Mini kit (Qiagen, Valencia, CA, USA), according to the manufacturer’s instructions. Fresh leaf tissue from each accession was used for each extraction and ground well using liquid nitrogen. DNA was resuspended in 100 μL water, and dilutions were made to 10 ng/μL followed by storage at either −20 °C or −80 °C. Randomly selected cDNA-SSR primer pairs were validated experimentally, and forward primers were synthesized by adding the M13 sequence to enable the addition of a fluorescent tail through the PCR amplification process [24]. PCR conditions included a hot-start at 95 °C for 10 min, followed by 10 cycles at 94 °C for 30 s, 60–50 °C for 30 s and 72 °C for 30 s, followed by 25 cycles at 94 °C for 30 s, 50 °C for 30 s, and 72 °C for 30 s, with a final elongation step of 72 °C for 10 min. PCR products were separated and visualized using the QIAxcel Gel Electrophoresis System (Qiagen).

### 3.6. Data Analysis

The amplified SSR loci were scored for 32 accessions. The total number of alleles (NA), major allele frequency (allele with the highest frequency) (*M_AF_*), observed heterozygosity (counting heterozygocity) (*H_O_*), expected heterozygosity (*H_E_*), number of genotypes (*N_G_*), and polymorphic information content (*PIC)* were calculated using PowerMarker and GenAlEx (version 6.5) [25]. 

The expected heterozygosity formula is as follows:

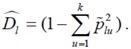
(1)


A closely related diversity measure is the polymorphism information content (*PIC*) [26]:

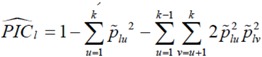
(2)

The cluster analysis of 32 accessions was carried out based onunweighted pair group method with arithmetic mean (UPGMA ) phylogenetic and uprooted tree construction, based on the “CS chord 1967” distance method [27] in powermarker

## 4. Conclusions

We developed 65 cDNA-SSR markers, which were used successfully to investigate the genetic diversity among 32 accessions of *Vicia stiva* subsp. *sativa*. Considering the relatively high *PIC* values (0.59 in average), cDNA-SSR in *Vicia sativa* subsp. *sativa* is suggested to be an informative genetic marker system, which can also be applied to population genetic studies and marker-assisted selection to mine and accumulate useful alleles to increase the agronomic potential of vetch varieties. 

## Supplementary Materials

Supplementary materials can be accessed at: http://www.mdpi.com/1420-3049/18/7/8376/s1.

## References

[1] Hueze V., Tran G., Baumont R. (2011). Common vetch (*Vicia sativa*). Feedipedia.

[2] Sullivan P. (2003). Overview of cover crops and green manures. ATTRA.

[3] Tate M., Ennenking D. (2006). Common vetch (*Vicia sativa* ssp. *sativa*): Feed or future food. Grain Legumes.

[4] Tate M.E., Enneking D. (1992). A mess of red pottage. Nature.

[5] Tate M.E., Rathjen J., Delaere I., Enneking D. (1999). Covert trade in toxic vetch continues. Nature.

[6] Thavarajah P., Thavarajah D., Premakumara G.A., Vandenberg A. (2012). Detection of Common Vetch (*Vicia sativa* L.) in Lentil (*Lens culinaris* L.) using unique chemical fingerprint markers. Food Chem..

[7] Uzun A., Gucer S., Acikgoz E. (2011). Common vetch (*Vicia sativa* L.) germplasm: correlations of crude protein and mineral content to seed traits. Plant Foods Hum. Nutr..

[8] Firincioglu H.K., Unal S., Erberkas E., Dogruyol L. (2010). Relationships between seed yield and yield components in common vetch (*Vicia sativa* ssp. *sativa*) populations sown in spring and autumn in central Turkey. Field Crop. Res..

[9] Firincioglu H.K., Erbektas E., Dogruyol L., Mutlu Z., Unal S., Karakurt E. (2009). Phenotypic variation in autumn and spring-sown vetch (*Vicia sativa* ssp.) populations in central Turkey. Span. J. Agric. Res..

[10] Mutz K.O., Heilkenbrinker A., Lonne M., Walter J.G., Stahl F. (2012). Transcriptome analysis using next-generation sequencing. Curr. Opin. Biotechnol..

[11] Ronaghi M. (2001). Pyrosequencing sheds light on DNA sequencing. Genome Res..

[12] Varshney R.K., Close T.J., Singh N.K., Hoisington D.A., Cook D.R. (2009). Orphan legume crops enter the genomics era!. Curr. Opin. Plant Biol..

[13] Luro F.L., Costantino G., Terol J., Argout X., Allario T., Wincker P., Talon M., Ollitrault P., Morillon R. (2008). Transferability of the EST-SSRs developed on *Nules clementine* (*Citrus clementina* Hort ex Tan) to other Citrus species and their effectiveness for genetic mapping. BMC Genomics.

[14] Kim T.S., Booth J.G., Gauch H.G., Sun Q., Park J, Lee Y.H., Lee K.G. (2008). Simple sequence repeats in *Neurospora crassa*: Distribution, Polymorphism and evolutionary inference. BMC Genomics.

[15] Wang Z., Fang B., Chen J., Zhang X., Luo Z., Huang L., Chen X., Li Y. (2010). De novo assembly and characterization of root transcriptome using Illumina paired-end sequencing and development of cSSR markers in sweet potato (*Ipomoea batatas*). BMC Genomics.

[16] Blanca J., Canizares J., Roig C., Ziarsolo P., Nuez F., Pico B. (2011). Transcriptome characterization and high throughput SSRs and SNPs discovery in *Cucurbita pepo* (Cucurbitaceae). BMC Genomics.

[17] Moe K.T., Chung J.W., Cho Y.I, Moon J.K., Ku J.H., Jung J.K., Lee J., Park Y.J. (2011). Sequence information on simple sequence repeats and single nucleotide polymorphisms through transcriptome analysis of mungbean. J. Integr. Plant Biol..

[18] Kaur S., Pembleton L.W., Cogan N.O., Savin K.W., Leonforte T., Paull J., Materne M., Forster J.W. (2012). Transcriptome sequencing of field pea and faba bean for discovery and validation of SSR genetic markers. BMC Genomics.

[19] Tanase K., Nishitani C., Hirakawa H., Isobe S., Tabata S., Ohmiya A., Onozaki T. (2012). Transcriptome analysis of carnation (*Dianthus caryophyllus* L.) based on next-generation sequencing technology. BMC Genomics.

[20] Yang T., Bao S.Y., Ford R., Jia T.J., Guan J.P., He Y.H., Sun X.L., Jiang J., Hao J., Zhang X., Zong X. (2012). High-throughput novel microsatellite marker of faba bean via next generation sequencing. BMC Genomics.

[21] Dangi R.S., Lagu M.D., Choudhary L.B., Ranjekar P.K., Gupta V.S. (2004). Assessment of genetic diversity in *Trigonella foenum-graecum* and *Trigonella caerulea* using ISSR and RAPD markers. BMC Plant Biol..

[22] Elahi E., Ronaghi M. (2004). Pyrosequencing: a tool for DNA sequencing analysis. Methods Mol. Biol..

[23] Margulies M., Egholm M., Altman W.E., Attiya S., Bader J.S., Bemben LA., Berka J., Braverman MS., Chen Y.J., Chen Z. (2005). Genome sequencing in microfabricated high-density picolitre reactors. Nature.

[24] Riley M. (1993). Functions of the gene products of *Escherichia coli*. Microbiol. Mol. Biol. Rev..

[25] Peakall R., Smouse P.E. (2012). GenAlEx 6.5: Genetic analysis in Excel. Population genetic software for teaching and research--an update. Bioinformatics.

[26] Botstein D., White R.L., Skolnick M., Davis R.W. (1980). Construction of a genetic linkage map in man using restriction fragment length polymorphisms. Am. J. Hum. Genet..

[27] Cavalli-Sforza L.L., Edwards A.W.F. (1967). Phylogenetic Analysis: Models and Estimation Procedures. Am. J. Hum. Genet..

