# Supplementary material for: Development of 65 Novel Polymorphic cDNA-SSR Markers in Common Vetch (Vicia sativa subsp. sativa) Using Next Generation Sequencing
Source: Molecules. 2013 Jul 16;18(7):8376–92. doi: 10.3390/molecules18078376 (PMC6270072; doi:10.3390/molecules18078376)

Supplementary Materials

Figure S1. SSR marker banding profile of 32 common vetch accessions using primer GBSSR-VSspS-181.

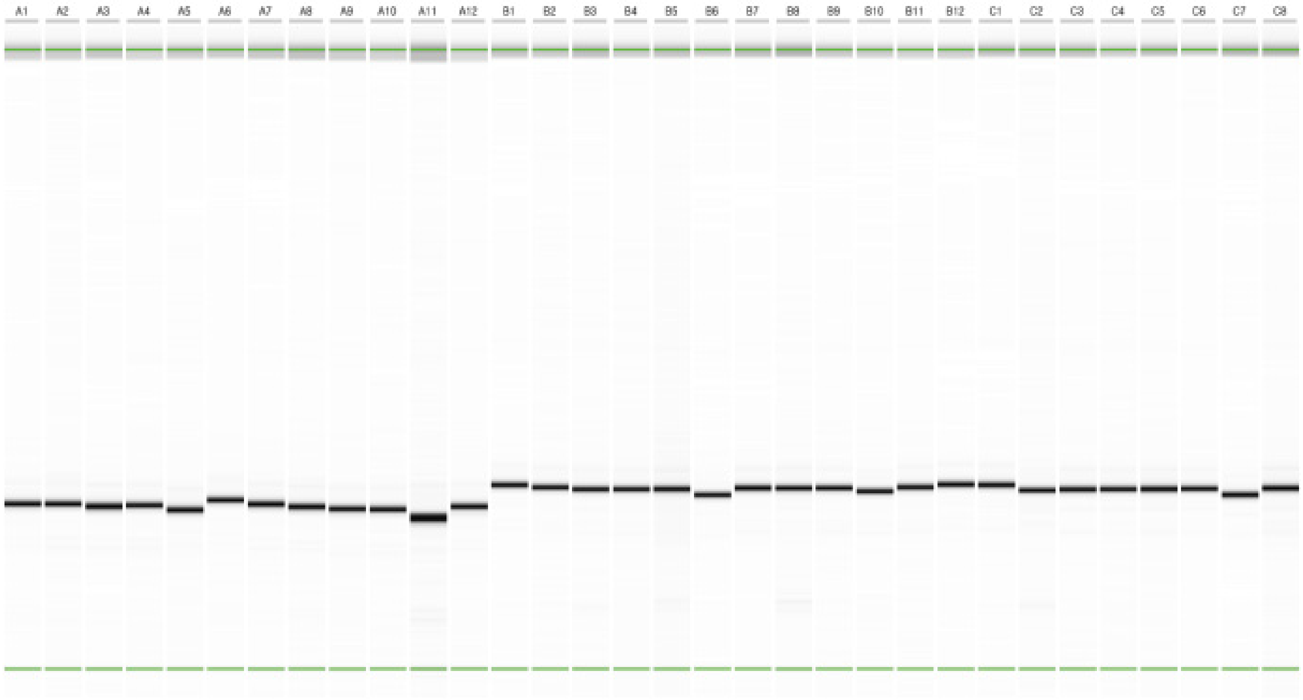

Supplement: Supplementary file 1 [file molecules-18-08376-s001.pdf]
